# Creation of Early Flowering Germplasm of Soybean by CRISPR/Cas9 Technology

**DOI:** 10.3389/fpls.2019.01446

**Published:** 2019-11-22

**Authors:** Jianan Han, Bingfu Guo, Yong Guo, Bo Zhang, Xiaobo Wang, Li-Juan Qiu

**Affiliations:** ^1^The National Key Facility for Crop Gene Resources and Genetic Improvement (NFCRI) and MOA Key Labs of Crop Germplasm and Soybean Biology (Beijing), Institute of Crop Science, Chinese Academy of Agricultural Sciences, Beijing, China; ^2^Crop Research Institute, Jiangxi Academy of Agricultural Science, Jiangxi, China; ^3^School of Plant and Environmental Sciences, Virginia Polytechnic Institute and State University, Blacksburg, VA, United States; ^4^College of Agronomy, Anhui Agricultural University, Hefei, China

**Keywords:** soybean, CRISPR/Cas9, gene editing, *E1*, *Agrobacterium*-mediated transformation

## Abstract

Soybean is an important economic crop and a typical short-day crop, sensitive to photoperiod, and has narrow geographical adaptative region, which limit the creation of transgenic materials and reduce the breeding efficiency of new varieties. In addition, the genetic transformation efficiency of soybean is lower than that of many other crops, and the available receptor genotypes are limited. In this study, *Agrobacterium*-mediated transformation were used to introduce the CRISPR/Cas9 expression vector into soybean cultivar Jack and generated targeted mutants of *E1* gene controlling soybean flowering. We obtained two novel types of mutations, 11 bp and 40 bp deletion at *E1* coding region, respectively, and frameshift mutations produced premature translation termination codons and truncated E1 proteins, causing obvious early flowering under long day condition. In addition, no off-target effects were observed by predicting and analyzing the potential off-target sites of *E1* targets. Significant decreased *E1* gene expression of two novel mutants showed that the truncated E1 protein disinhibited *GmFT2a*/*5a* and increasing *GmFT2a*/*5a* gene expressions resulted obvious early flowering. Homozygous trans-clean mutants without T-DNA elements were also obtained and showed early flowering under long day condition. The photo-insensitive soybean transformation receptor we created laid a foundation for breeding excellent transgenic receptors suitable for high latitudes.

## Introduction

Soybean is rich of protein and oil and has high economic value. With the increasing demand for soybean globally, it is urgent to clarify gene function, and accelerate functional gene research and breeding speed for increasing yield and improving quality. In recent years, CRISPR (Clustered regular interspaced short palindromic repeat)/Cas9 (CRISPR-associated) provides an effective method for targeted genome editing and gene function research, and supplies a new idea for reverse genetics research. CRISPR/Cas9 system includes gene knock out, knock in, multiple genes and sites editing, large fragment deletion and replacement (Gratz et al., 2013; Shan et al., 2013; Feng et al., 2014; Gratz et al., 2014; Zhou et al., 2014). Feng et al. (2013) and Mao et al. (2013) firstly proved that CRISPR/Cas9 system could be used to target genome editing in crop by introducing site-directed mutations in specific genes in *Arabidopsis* and rice. Subsequently, CRISPR/Cas9 technology has been widely used in several species including rice (Shan et al., 2013), wheat (Upadhyay et al., 2013), cotton (Gao et al., 2017), maize (Chen et al., 2018), *Arabidopsis* (Li et al., 2013), tobacco (Gao et al., 2015) and barley (Kapusi et al., 2017). Jacobs et al. (2015) first used CRISPR/Cas9 technology to knock out the green fluorescent protein gene (GFP), and produced targeted editing for nine soybean endogenous genes. Then CRISPR/Cas9 technology began to be widely applied in soybean (Cai et al., 2015; Jacobs et al., 2015; Li et al., 2015; Michno et al., 2015; Sun et al., 2015; Du et al., 2016; Tang et al., 2016; Cai et al., 2018; Li et al., 2019). Therefore, CRISPR/Cas9 is an effective tool for soybean targeted genome editing, providing a theoretical and technical basis for further research on soybean genome, as well as improving the breeding efficiency and accelerating the breeding process.

CRISPR/Cas9 system relies on transformation technology. However, soybean transformation efficiency is lower than other crops (Du et al., 2016), and receptor genotype dependence is a major limiting factor. The transformation efficiency of soybean is usually low, and the varieties suitable for soybean genetic transformation are very few (Donaldson and Simmonds, 2000; Guo et al., 2015). At the same time, soybean is a typical short-day crop and sensitive to photoperiod, which limits the geographical cultivated region (Xu et al., 2013; Wang et al., 2016), excellent varieties creation and breeding efficiency.

Soybean varieties are adapted to different latitudes and different photoperiods, so they need to have a series of photo-insensitivity. Photoperiod is the key meteorological factor that determines flower bud differentiation and adaptation to different ecological regions (Camara et al., 1997). The wide adaptability to different latitudes in soybean is controlled by some major genes and QTLs (Watanabe et al., 2012). At present, 11 genes have been identified to be related to soybean growth period (*E1*-*E10*, *J*) (Bernard, 1971; Buzzell, 1980; Buzzell and Voldeng, 1980; Mcblain and Bernard, 1987; Mcblain et al., 1987; Ray et al., 1995; Cober et al., 1996; Bonato and Antonio, 1999; Cober et al., 2010; Kong et al., 2014; Lu et al., 2017; Yue et al., 2017). Among these genes, *E1* has the greatest influence on soybean growth period with the most inhibitory of flowering and is considered as the most important gene controlling soybean growth period (Bernard, 1971; Cober et al., 1996; Watanabe et al., 2012; Xia et al., 2012) and is also a main selection locus in soybean breeding (Xia, 2017). *E1*, located at centromere of chromosome 6, is a unique transcription to legumes. It has bilateral nuclear location signal and DNA binding site, and is a flowering inhibitor related to the B3 domain (Xia et al., 2012). Seven alleles have been identified for *E1*. Its amino acids changes in the nuclear location signal region resulted in its protein distribution changing. E1 protein distributes in the nucleus, while e1 protein distributes in the nucleus and cytoplasm (Xia, 2013). *E1* gene expression is closely related to the length of day light. Under long day condition (LD), *E1* delays flowering by negative regulation of *GmFT2a*/*5a*, while its recessive alleles have early flowering time by disinhibiting *GmFT2a*/*5a* expression (Zhang et al., 2016). *E1* gene expression was significantly inhibited under short day condition which is the main factor of photoperiod sensitivity in soybean (Lü, 2015).

In this study, we used CRISPR/Cas9 system and *Agrobacterium*-mediated transformation to introduce the CRISPR/Cas9 expression vector into soybean cultivar Jack, knocked out *E1* gene and analyzed the effect of novel *E1* mutants to soybean flowering. It provides materials for breeding early flowering receptors, promotes the development of soybean genetic improvement, provides a basis for efficient soybean genetic transformation, and establishes an important guide for soybean gene function research, molecular breeding and variety layout.

## Materials and Methods

### Plant Materials and Growth Conditions

Soybean cultivar Jack was used for genetic transformation receptor. Jack (wild type as a control) and all seeds harvested from T_0_ plants obtained through CRISPR/Cas9 and *Agrobacterium*-mediated transformation were planted on June 22, 2017 at the Shunyi Experimental Station of the Institute of Crop Sciences, Chinese Academy of Agricultural Sciences. T_2_ homozygous mutants were planted in greenhouse under long day conditions (LD, 16 h/8 h, light/dark) and short-day conditions (SD, 12 h/12 h, light/dark) at 28, 70% relative humidity. The flowering time of each plant from seedling emergence to R1 stage (the first flower appeared on any node of the main stem) was recorded according to Fehr et al. (1971). Data analysis was performed by using Microsoft Excel, then adopted one-way analysis of variance. P < 0.01 was considered statistically significant.

### CRISPR/Cas9 Expression Vector Construction

**Figure 1:**
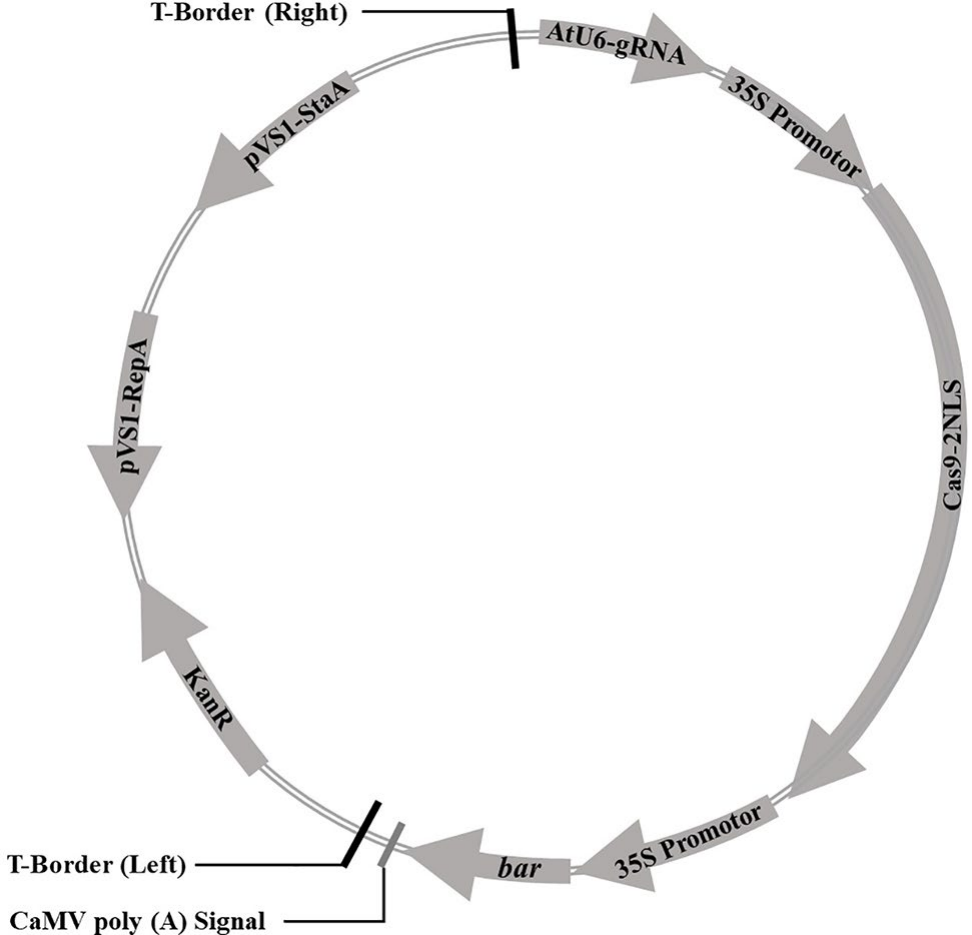
The vector pBSE401 used for CRISPR/Cas9-mediated genome editing. AtU6, *Arabidopsis* U6 promotor; gRNA, guide RNA; 35S promotor, CaMV 35S promotor; Cas9, codon-optimized Cas9; NLS, nuclear location signal; *bar*, selective marker gene; KanR, Kanamycin resistance gene; pVS1-RepA, pVS1 replication origin; pVS1-StaA, pVS1 stability function.

Cas9 sequence was optimized for the codon-optimized for dicotyledons and connected with CaMV 35S promoter at downstream, then assembled with sgRNA driven by *Arabidopsis* U6 promoter to construct a plasmid vector containing both sgRNA and Cas9. The bar gene as a selective marker was driven by CaMV 35S promoter. The CRISPR/Cas9 expression cassette was shown in **Figure 1**. The sequences were synthesized by Shanghai Sangon Biotech (Shanghai, China). The soybean endogenous gene *E1* (*Glyma*.*06G207800*) sequence and its information were downloaded from the Phytozome database (www.phytozome.net/). The optimal sgRNA sequences (20 bp) with the G as the first base were designed using online tool (www.genome.arizona.edu/crispr/CRISPRsearch.html). The base G was added at the 5′ end artificially if the first base was not G (Ran et al., 2013). The online website (www.rgenome.net/cas-offinder/) was used to evaluate off-target effects. Two sgRNAs for the *E1* gene we selected were named as *E1*-SP1 (5′-CCCTTCAGATGAAAGGGAGCAG-3′) and *E1*-SP2 (5′-CCACCATATGCGAAGCCTCTAA-3′) respectively. Primers containing either of two sgRNAs were synthesized by Shanghai Sangon Biotech (**Supplementary Table 1**). Using overlapping polymerase chain reaction (PCR), two sgRNAs were cloned into pCBC plasmid. PCR products containing two sgRNAs were digested and inserted into the pHSE401 plasmid vector to construct the CRISPR/Cas9 expression vector containing Cas9 and sgRNA (**Supplementary Figure 1**). And then the CRISPR/Cas9 expression vector was transformed into *E. coli* Trans1 T1 (TransGen Biotech) used for soybean genetic transformation.

### *Agrobacterium*-Mediated Soybean Transformation

The CRISPR/Cas9 expression vector was transferred into *Agrobacterium* strain EHA105 by electroporation. Soybean cultivar Jack was used for tissue culture and soybean transformation. Soybean genetic transformation procedure was referred to the described method in our laboratory (Guo et al., 2015) and made appropriate modification. The healthy soybean seeds were sterilized with chlorine for 16 h and germinated 5 d to prepare explants. Explants were submerged in agrobacterium suspension adding 0.02% surfactant (Silwet L-77) and wounded by ultrasonic treatment. After infection for 30–40 min, explants were co-cultured for 3 d. After the tissue culture (resistant bud induction, shoot elongation and rooting), transgenic plants were regenerated from explants. At the stage of resistant bud induction and shoot elongation, we added 10 mg L^-1^ and 6 mg L^-1^ glufosinate (Sigma-Aldrich, USA) respectively into culture medium to screen positive transformed cells.

### Sequence Analysis of the Transgenic Plants

Total genomic DNA was extracted from every leaf sample following the modified cetyltrimethylammonium ammonium bromide (CTAB) protocol in the T_0_, T_1_ and T_2_ generation (Saghai-Maroof et al., 1984). To determine the types of mutation at target sites, we used specific primers (**Supplementary Table 2**) containing target sites in *E1* gene and genomic DNA as the template to amplify and analyze the target sites sequence. PCR products were detected by 1% agarose gel electrophoresis and then sequenced. Three types of mutations were identified by sequence peaks. Heterozygous mutations showed chaotic peaks after the target site, while wild types and homozygous mutations showed single peaks at the target. The sequences of homozygous mutations were aligned with wild types to further determine the variation of target. To screen and obtain *E1* targeted mutants without transgenic elements, PAT/Bar test strip was used to identify the selective marker *bar* gene. Two pairs of primers (**Supplementary Table 2**) were used to determine sgRNA/Cas9 on T-DNA elements by PCR.

### Real-Time Quantitative PCR Analysis of Gene Expression

Expression levels of *E1* and *GmFT2a*/*5a* in wild type plants and T_2_ homozygous mutants were analyzed under LD and SD conditions, respectively. Every 5-day interval after 10 days after emergence (DAE), at 10 am (4 h after light), the trifoliate leaves were sampled from plants with different genotypes under LD and SD conditions. Total RNAs were extracted using TransZol Up Plus RNA Kit (TransGen Biotech). For reverse transcription, the first-strand cDNA synthesis was performed using the TransScript First-strand cDNA Synthesis SuperMix Kit (TransGen, China). For qRT-PCR, gene expressions were examined using cDNA templates on an Applied Biosystems 7300 Real-Time PCR System. The relative gene expression levels followed the method (Pfaffl, 2001). The mRNA level of *GmActin* (*Glyma18g52780*) was used as a reference for normalization. Specific primers we used in this study were list in **Supplementary Table 2**. Three biological replicates were used for each gene.

## Results

### CRISPR/Cas9-Mediated Mutations

**Figure 2:**
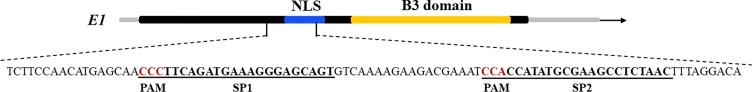
Gene structures of *E1* with target sites. The underlined bases indicate the target sequences, and the red letters indicate PAM sequences.

**Figure 3:**
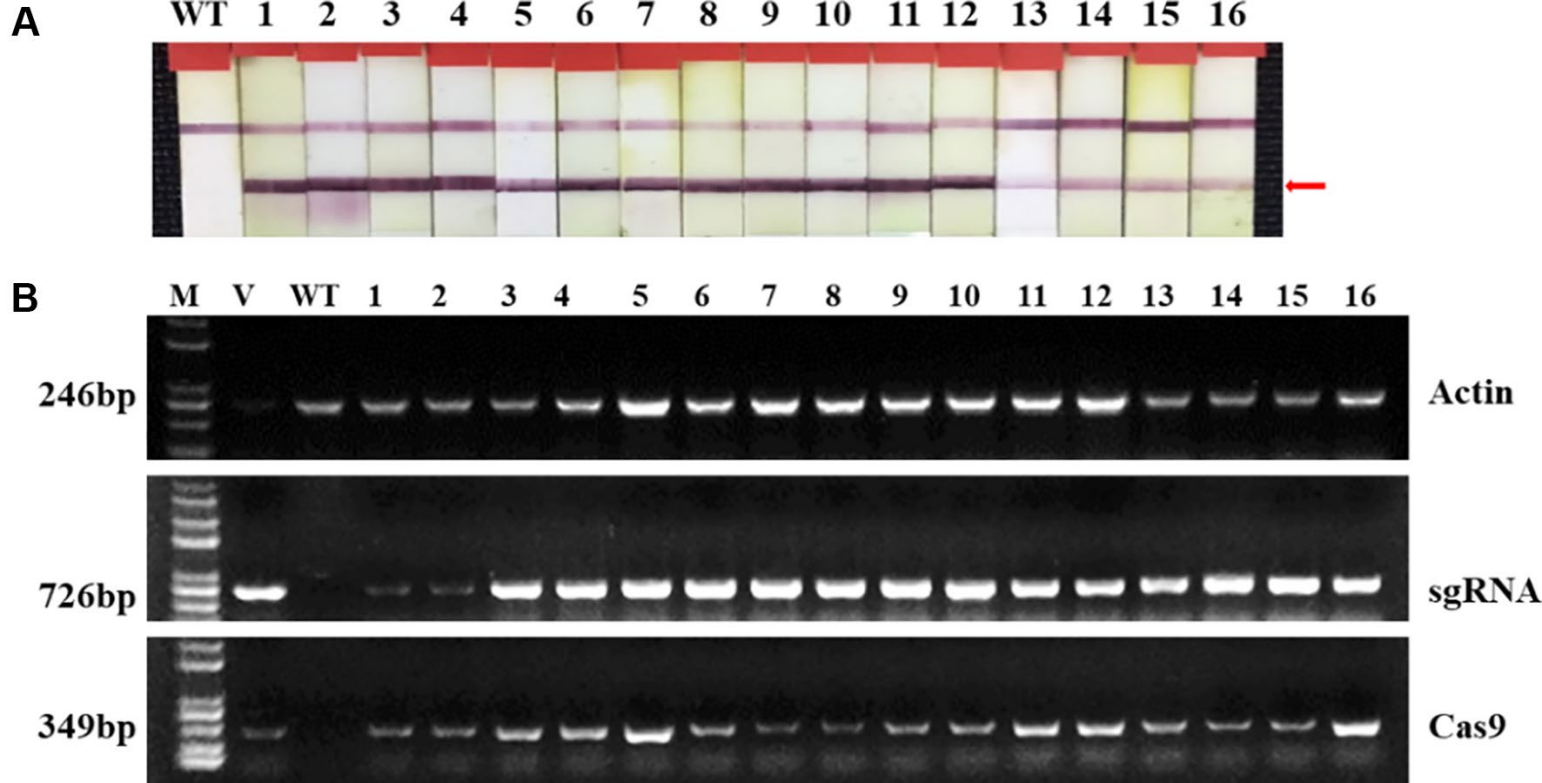
Identifying transplants in T_0_ generation. **(A)** Detection of the selectable marker gene bar by PAT/Bar test strip. The red arrowhead indicates that bar gene is positive. **(B)** Gel image of PCR products for T-DNA regions. Cas9, part of the Cas9 coding sequence. sgRNA, region from the U6 promoter to the downstream vector sequence spanning the sgRNA. *GmActin* was used as a normalization control. V: plasmid of the vector in transformation. WT, wild type soybean plants. Labels 1-16, individual mutant lines.

Two targets for the *E1* gene (named *E1*-SP1 and *E1*-SP2, respectively) were designed (**Figure 2**), and the CRISPR/Cas9 expression vector were transferred into the soybean cultivar Jack by *Agrobacterium*-mediated genetic transformation to knock out soybean endogenous gene *E1*. The whole genome DNAs of transformed plants were used for PCR and sequence analysis. Combining with PAT/Bar test strip detection, 16 T-DNA positive plants were obtained (**Figure 3**), of which 12 plants had heterozygous mutations at target sites (**Supplementary Figure 2**). Then, all seeds derived from 9 heterozygous T_0_ generation were planted in the Shunyi Station of the Chinese Academy of Agricultural Sciences under LD conditions (3 of 12 T_0_ plants had no seed) and the types of mutation at target sites were determined. Six out of nine lines had three types of mutations: homozygous mutation, heterozygous mutation and non-mutation (**Table 1**). The rest three T_1_ lines didn’t show mutation, and site-directed mutagenesis of T_0_ generation didn’t inherit to its progeny.

**Table 1:** CRISPR/Cas9-mediated targeted mutants of *E1* in the T_1_ generation.

T_1_ generation	No. of plants sequenced	No. of homozygous plants	No. of heterozygous plants	No. of no mutant plants
L1	4	0	3	1
L7	2	2	0	0
L9	4	2	2	0
L10	6	0	4	2
L11	10	0	8	2
L16	9	6	3	0
Total	35	10	20	5

Two types of homozygous mutations were detected at the target sites (named *e1*-1 and *e1*-2, 11 bp deletion and 40 bp deletion, respectively) in three lines including L7 (*e1*-1), L9 (*e1*-2) and L16 (*e1*-1). All of them were frameshift mutations, resulting in premature translation termination codons (**Supplementary Figure 3**). The 11 bp deletion of *e1*-1 created a truncated protein encoding 79 amino acids, and caused the absence of all the B3 domains while keeping part of nuclear location signal. The 40 bp deletion of *e1*-2 created a truncated protein encoding 88 amino acids, and caused the absence of neither B3 domain or nuclear location signal (**Supplementary Figure 4**).

### Potential Off-Target Analysis

In order to determine whether the CRISPR/Cas9 expression vector we used to have variations at potential off-targets and avoid the possibility of potential off-targets effecting on phenotype, by using online website (http://cbi.hzau.edu.cn/crispr/), four most potential off-target sites at the two targets (*E1*-SP1 and *E1*-SP2) were selected. Every potential off-target site mismatched 2-4 bases with the *E1* target sequences (**Table 2**). Using specific primers of potential off-target sites (**Supplementary Table 2**) and genome DNAs of 35 T_1_ mutants as templates for PCR, we didn’t detect variation at four potential off-targets. The sequence comparison analysis shown in **Supplementary Figure 5** indicated that CRISPR/Cas9 expression vector had specific edits in *E1*-SP1 and *E1*-SP2 targets.

**Table 2:** Potential off-target analysis at the two target sites of *E1* in the T_1_ generation.

Target	Potential target	Physical position	Target sequence	No. of mismatch	Position
*E1*-SP2	OFF-1	*Glyma04g24640* 4: -28294190	CCACCATATGtGAAGCCTCcAAC	2	exon
	OFF-2	*Glyma10g33050* 10: -41421475	CCTCCATATGaGAAGCCaCcAcC	4	exon
*E1*-SP1	OFF-3	*Glyma17g06520* 17: -4658911	CCATTaAaATGAAAGGaAGCAGc	4	intron
	OFF-4	*Glyma19g02555* 19: -2360418	CTTTTCAGATGAAgtGGAGCAGc	3	exon

Mismatched bases are shown in lowercase red letters.

### Inheritance Analysis and Phenotype Identification

**Figure 4:**
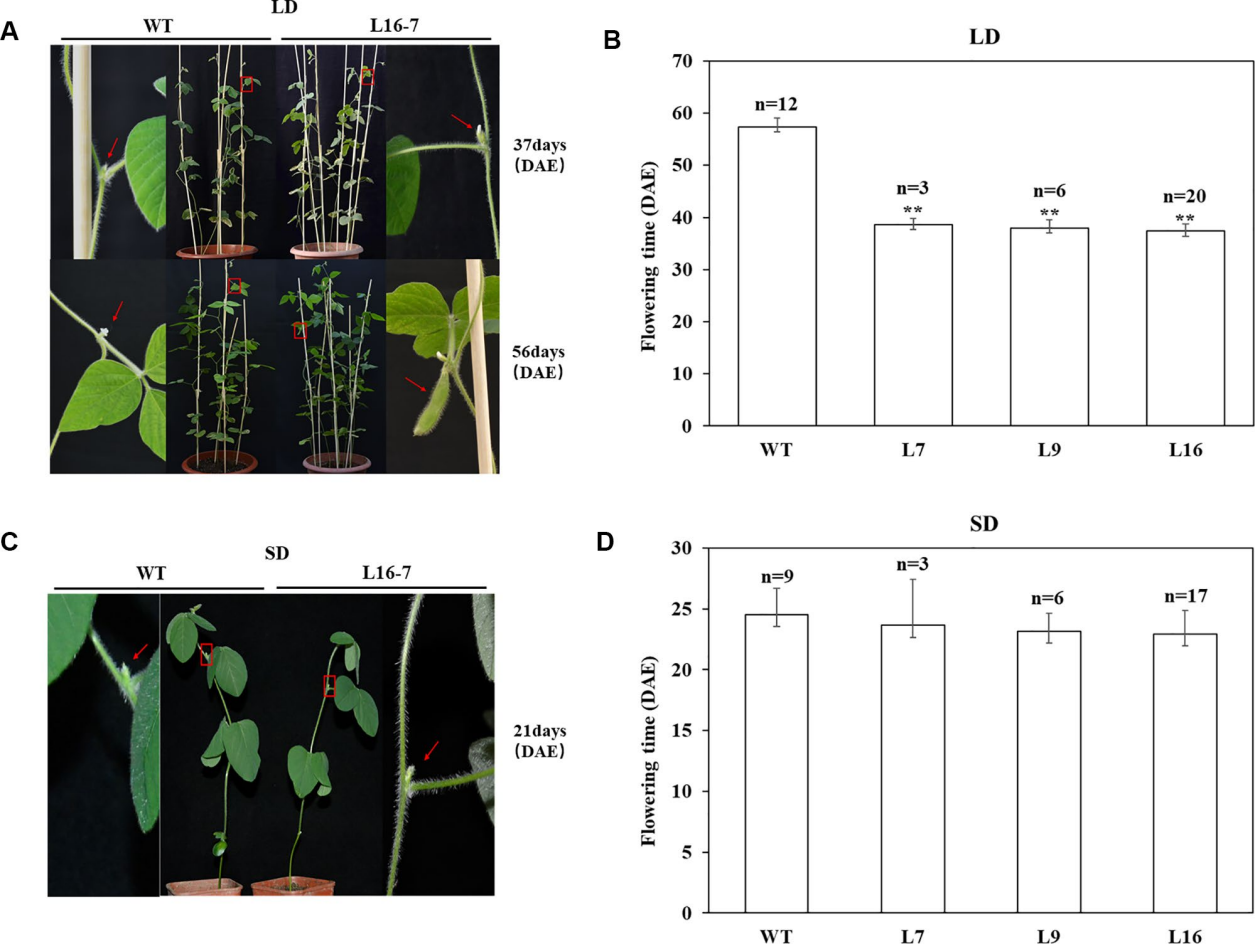
CRISPR/Cas9-induced *E1* mutants flowering time under both LD and SD conditions. **(A)** Phenotypes of wild type (WT, Jack) and homozygous T_2_ mutant under LD condition, respectively. Top panel, WT did not have floral buds when T_2_ mutant was flowering. Bottom panel, T_2_ mutant produced the pods when WT was flowering. Red box, magnified view. **(B)** Flowering time of WT and homozygous T_2_ mutants under LD condition. **(C)** Phenotypes of wild type (WT, Jack) and homozygous T_2_ mutant under SD condition, respectively. **(D)** Flowering time of WT and homozygous T_2_ mutants under SD condition. n, exact numbers of individual plants identified. **, homozygous T_2_ mutants exhibit significant early flowering time (P < 0.01). The flowering time is shown as the mean values ± standard deviation.

To identify whether the mutations at the target of the homozygous T_1_ mutants could inherited to T_2_ generation stably, T_2_ seeds derived from homozygous mutants of T_1_ lines (L7, L9 and L16) were planted under LD and SD conditions, respectively (**Table 3**). Sequence analyses of 28 T_2_ individuals indicated that CRISPR/Cas9-mediated mutagenesis of *E1* gene could be stably inherited from T_1_ generation to T_2_ generation and maintained the same type of variation. To analyze the flowering time accurately, R1 period of all above T_2_ plants were recorded under LD and SD conditions respectively. Under LD condition, when T_2_ homozygous mutants were flowering, the wild type plants had not shown any flower buds. When wild type plants were flowering, T_2_ homozygous mutants had obvious pods (**Figure 4A**). The recorded flowering dates showed that flowering time of mutants was significantly earlier than that of wild type plants (P < 0.01). The average flowering time of wild type plants were 57 d, while the average flowering time of T_2_ homozygous mutants derived from three different lines (L7, L9 and L16) were 39 d, 38 d and 37 d, respectively (**Figure 4B**). However, under SD condition, when the T_2_ homozygous mutants were flowering, the wild type plants were flowering (**Figure 4C**) and we didn’t observe significant difference between wild type plants and mutants. The average flowering time of wild type plants was 25 d, while the average flowering time of T_2_ homozygous mutants derived from L7, L9 and L16 were 24 d, 23 d, and 23 d, respectively (**Figure 4D**). Under natural condition, there were no significant differences in plant height, node number and branch number between mutants and wild type plants (**Supplementary Figure 6**).

**Table 3:** T_2_ homozygous mutants under LD and SD conditions.

T_1_ generation	Mutation type	T_2_ generation	
		No. of plants in LD	No. of plants in SD
L7	40 bp delete	3	3
L9	11 bp delete	6	6
L16	40 bp delete	19	17

### Gene Expression Analysis of *E1*/*GmFT2a*/*GmFT5a*

**Figure 5:**
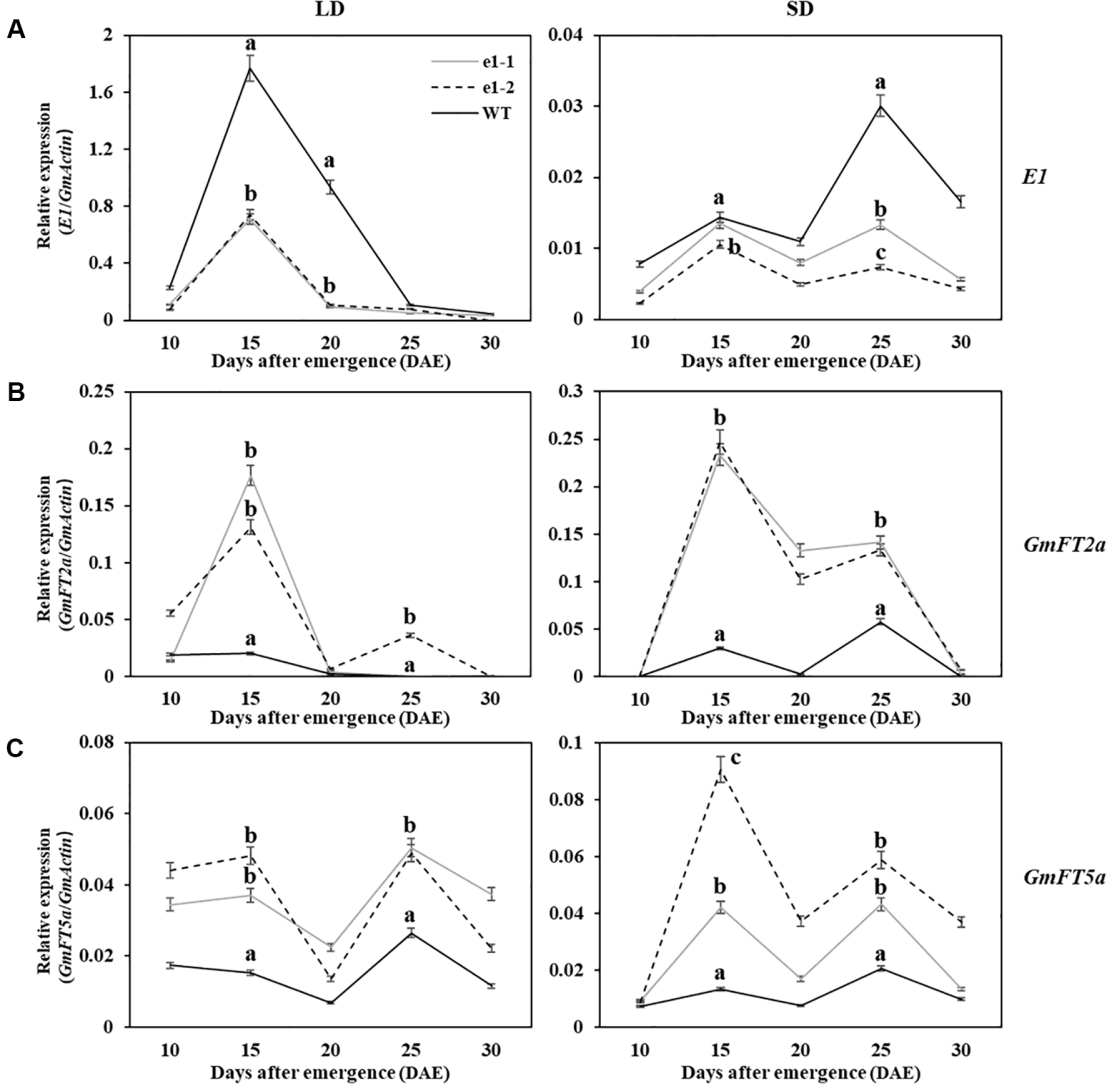
Expression analyses of *E1/GmFT2a/5a* in WT plants and mutants under LD and SD conditions. **(A)** Expression analysis of *E1* under LD and SD conditions. **(B)** Expression analysis of *GmFT2a* under LD and SD conditions. **(C)** Expression analysis of *GmFT5a* under LD and SD conditions. The relative expression levels are showed as the mean values ± standard deviation, which was calculated from three biological replicates. a, b and c indicate significant differences (P < 0.01).

Studies had shown that negative regulation model between *E1* and *GmFT2a*/*5a* was closely related to flowering time under LD and SD conditions (Kong et al., 2014; Xia et al., 2012). In order to clarify the correlation between the expression of *E1* and *GmFT2a*/*5a* with flowering time of mutants, RNA was extracted from trifoliate leaves in two types of mutations of L7, L9 and L16 lines under LD and SD conditions at 10 am every fifth day after 10 DAE for five times (**Figure 5**). Wild type plants and mutants showed the similar expression patterns for each of three genes (*E1*/*GmFT2a*/*5a*) under LD or SD condition, but different expression levels. Under LD condition, *E1* had the highest expression level at 15 DAE both in mutants and wild type plants, but significantly lower *E1* expression level in mutants was observed at 15 and 20 DAE (P < 0.01). *GmFT2a*/*5a* exhibited two expression peaks at 15 DAE and 25 DAE both in mutants and wild type plants, but there were significantly higher expression levels in mutants at 15 and 25 DAE (P < 0.01). Under SD condition, all the three genes (*E1*/*GmFT2a*/*5a*) showed bimodal expression patterns and the expression peaks appeared at 15 DAE and 25 DAE, respectively. However, *E1* gene expression was significantly lower and almost no expression than that under LD condition.

### Trans-Clean Mutants Without T-DNA Elements

**Figure 6:**
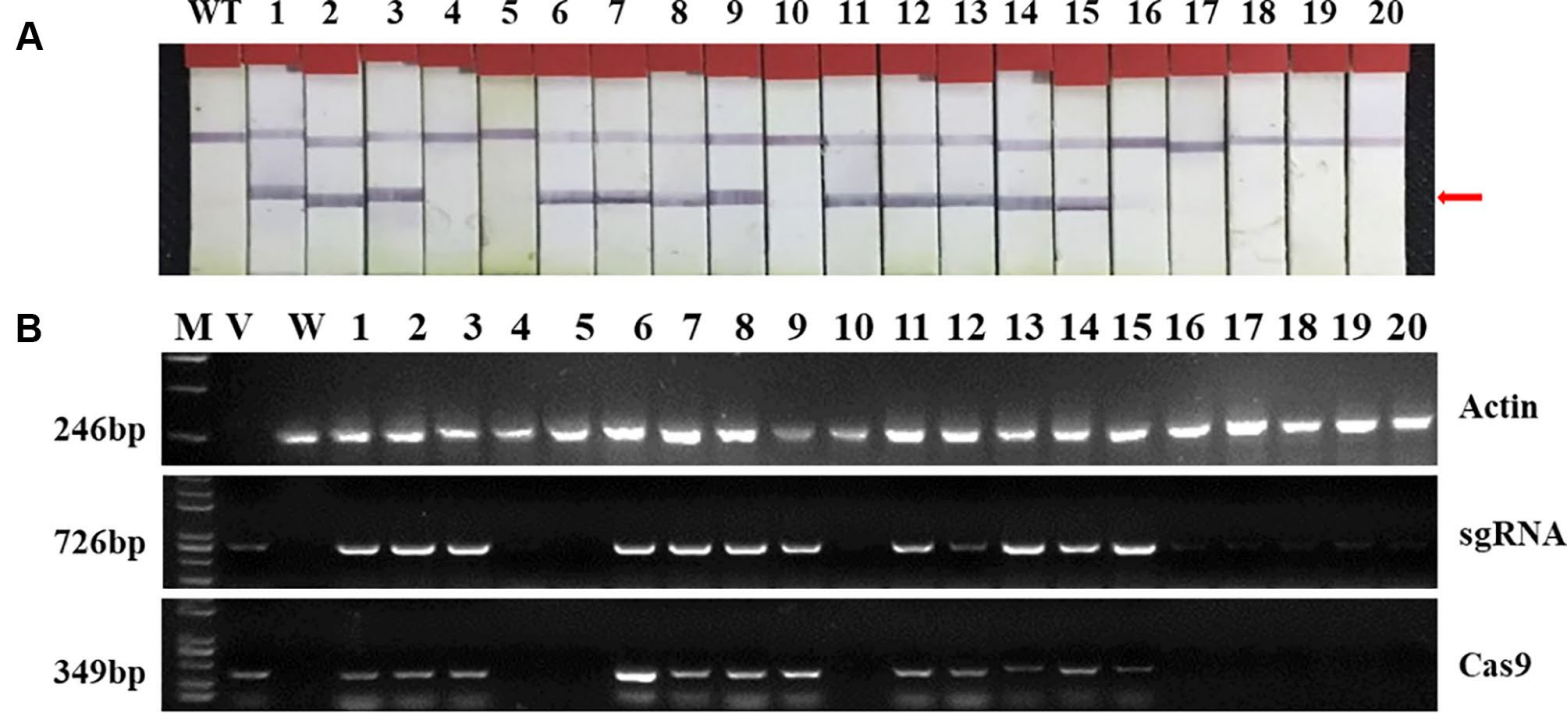
Identifying of trans-clean mutants. **(A)** Detection of the selectable marker gene bar by PAT/Bar test strip. The red arrowhead indicates that *bar* gene is positive. **(B)** Gel image of PCR products for T-DNA elements. Cas9, part of the Cas9 coding sequence. sgRNA, region from the U6 promoter to the downstream vector sequence spanning the sgRNA. *GmActin* was used as a normalization control. V: plasmid of the vector in transformation. WT, wild type. Labels 1-20, individual mutant lines.

To obtain novel soybean germplasm with homozygous mutation but without T-DNA elements, PAT/Bar test strip was used to identify the selective marker gene *bar* firstly and PCR strategy was used to exam sgRNA/Cas9 on T-DNA by using specific primers (**Supplementary Table 2**). Among three T_1_ lines, only L7 didn’t show T-DNA elements, and its 50 progenies were all free of T-DNA. Only 11 out of 211 homozygous mutants were free of T-DNA in T_2_ generation derived from L9 and L16 (**Table 4**, **Figure 6**).

**Table 4:** Transgene-clean homozygous mutants from T_1_ and T_2_ generations.

T_1_ generation	T-DNA	T_2_ generation	
		No. of plants identified	No. of trans-clean plants
L7	free	50	50
L9	positive	5	2
L16	positive	206	9
Total	–	261	61

## Discussion

Soybean is an important source of protein and oil for human. Therefore, gene function exploration and utilization are of great significance to increase yield and improve seed quality. However, soybean is a short-day crop, sensitive to photoperiod and had narrow geographical cultivated regions. In *Agrobacterium*-mediated genetic transformation, soybean transformation efficiency is lower than many other crops, and depends on genotypes used for transformation. At present, the soybean receptors with higher transformation efficiency are limited. Therefore, its sensitivity to photoperiod and difficulty in developing transgenic offspring have significantly restricted the improvement of breeding efficiency of new varieties and germplasm. Therefore, it is important to study soybean growth-related genes, breed and create new soybean germplasm with wide adaptability to different latitudes. Among soybean growth-related genes, *E1* has the greatest impact on growth period and has the most obvious photoperiod response, so it is considered as a major gene for controlling soybean flowering (Bernard, 1971; Cober et al., 1996; Watanabe et al., 2012; Xia et al., 2012).

In this study, we screened and obtained early flowering homozygous mutants without transgenic elements of soybean endogenous *E1* gene by CRISPR/Cas9 system. The random integration of foreign genes in plant chromosome may lead to destruction of plant endogenous gene, gene silencing and other undesirable phenomena, which caused a great controversy about the biosecurity of genetically modified organisms (Napoli et al., 1990). Therefore, the promotion and application of transgenic crops are greatly restricted (Zhang et al., 2011). CRISPR/Cas9 system affords an effective method to eliminate undesirable phenomena in the transformation by editing the target gene accurately. At the same time, the transgenic elements in CRISPR/Cas9 system such as Cas9 and other selective markers can be lost by progeny separation or selfing and obtain trans-clean mutants (Cai et al., 2018; Chen et al., 2018). Besides, off-target effects could be reduced by selecting target sequences specifically (Xie et al., 2014; Xu et al., 2015). As an effective genome editing tool, CRISPR/Cas9 technology has been widely used in many crops and there are also many applications in soybean. Jacobs et al. (2015) reported the site-directed mutagenesis in soybean by using CRISPR/Cas9 technology firstly, which laid a good foundation for soybean genome editing. Many studies have also successfully obtained mutants without transgenic elements in many crops by CRISPR/Cas9. Haun et al. (2014) generated a high oleic acid content soybean variety without transgenic components and improved the quality of soybean. Cai et al. (2018) obtained trans-clean soybean homozygous mutants with late flowering time by using CRISPR/Cas9 technology to knock out *GmFT2a*.

*E1* is a unique transcription factor to legumes and its B3 domain and nuclear location signal are important modules controlling soybean flowering (Xia et al., 2012). *E1* gene includes at least 7 allelic natural variations with *E1* and *e1*-*as* as the two basic genotypes. *e1*-*as* has a single missense mutation at the region of nuclear location signal. This one amino acid change led to the cell localization change and e1 protein distribution in the nucleus and cytoplasm at the same time. However, *e1*-*as* is a leaky allele and has partially function of delaying flowering (Xia, 2013). The other three nonfunctional alleles are *e1*-*fs*, *e1*-*nl* and *e1*-*b3a*. *e1*-*fs* has 1 bp deletion in the B3 domain, and this frameshift mutation resulted in a truncated protein encoding 41 amino acids. *e1*-*nl* is a null allele and all the *E1* gene is deleted. *e1*-*b3a* allele has 3 SNPs and 2 bp deletions in the B3 domain resulting in frameshift mutation (Zhai et al., 2015). *e1*-*re* and *e1*-*p* have variations at 5′UTR, but the flowering mechanism of these two alleles is unclear (Tsubokura et al., 2014). The two novel types of homozygous germplasm we obtained had 11 bp deletion and 40 bp deletion in the coding region, respectively, resulting in pre-terminate codons and truncated E1 proteins. *e1*-1 coded a 79-aa and deleted all B3 domain. *e1*-2 coded an 88-aa and completely deleted the nuclear location signals and B3 domain.

In addition, we compared the flowering time (R1) of *E1* mutants and wild-type. Under LD condition, the two types of homozygous mutants showed about 20 days earlier flowering time than wild type plants. The flowering time of the wild type plants was about 57 days and the average flowering time of mutants was only about 38 days. Studies have shown that the flowering time of cultivars carrying *E1*, *e1*-*as*, *e1*-*fs* and *e1*-*nl* were 70 d, 50 d, 30 d and 30 d under LD condition, respectively (Xia et al., 2012). In our study, the average flowering time of novel germplasm was about 38 days which was like the natural alleles *e1*-*fs* and *e1*-*nl*, indicating that the new *E1* mutants we obtained by CRISPR/Cas9 system may have the same mechanism for flowering as natural alleles. Studies have shown that nonsense mediated mRNA decay (NMD) reduces mRNAs with premature translation termination codons (PTCs) by down-regulating gene expression, and reduces its encoding truncated protein production (Baker and Parker, 2004; Maquat, 2004; Conti and Izaurralde, 2005; Maquat, 2005). The two mutants obtained in our study had bases deletion, leading to PTCs and truncated E1 protein. We speculated that NMD resulted in decreased *E1* gene expression in mutants compared with wild type plants.

Soybean is a typical short-day crop and its flowering time is closely related to the length of day light. The expression of *E1* was negatively correlated with *GmFT* (*GmFT2a*/*5a*) and controlled the *GmFT2a*/*5a* that functionally coordinated each other (Xia et al., 2012). In this study, compared with wild type plants, *E1* gene sequence of mutants caused not only truncated E1 protein but also significantly decreased gene expressions. Meanwhile, *GmFT2a*/*5a* expressions of mutants were significantly increased due to decreased *E1* gene and appeared earlier flowering time. In order to identify whether the decreased *E1* gene expression had influence on its homologous gene *E1*-*L* (*Glyma04g24640*.*1*/*Glyma18g22670*) and avoid its potential effect on the flowering time, we analyzed the *E1*-*L* gene expression. Result showed that there was no obvious difference on *E1*-*L* gene expression between mutants and wild type plants (**Supplementary Figure 7**).

*E1* is the most important gene controlling flowering and is also the major determinant to short-day crops. Photosensitivity in soybean reduced the flowering time, leading to early maturation and low yield in low latitude areas. Under SD condition, long juvenile trait can ensure enough growth period in soybean (Hartwig and Kiihl, 1979). *J* (*Glyma*.*04G050200*) is a transcriptional suppressor of *E1* gene and its recessive allele, *j*, delaying flowering time by disinhibit *E1*. The amplitude to delay flowering time is determined by different *E1* alleles (Lu et al., 2017). Furthermore, studies have shown that long-juvenile trait in soybean may be controlled by other genes (Ray et al., 1995, Valéria et al., 2000, Yue et al., 2017). Therefore, mutants we obtained not only provide soybean new receptor for high latitudes, and can also change the function of *J* at low latitudes, which may provide soybean high yield potential. In addition, it provides materials and theoretical basis for identifying other genes controlling long-juvenile trait and studying further the flowering regulation pathways. Compared with cross breeding, using CRISPR/Cas9 technology to create early flowering soybean material has the advantages significantly short breeding period with high efficiently.

## Conclusion

Our study provides materials support for breeding early-maturing transgenic receptors suitable for high latitudes and contributes to the soybean introduction. The photo-insensitive soybean transformed receptors could improve the soybean genetic development and contribute to efficient soybean genetic transformation. It offers important guidance for molecular breeding, soybean gene function research and variety development.

## Data Availability Statement

The datasets generated for this study are available on request to the corresponding author.

## Author Contributions

JH performed the experiments, analyzed the data, and wrote the manuscript. BG, YG, and XW provided advice on experimental implementation. XW, BZ, and L-JQ supervised the project, reviewed and revised the manuscript.

## Conflict of Interest

The authors declare that the research was conducted in the absence of any commercial or financial relationships that could be construed as a potential conflict of interest.
