# Supplementary material for: Creation of Early Flowering Germplasm of Soybean by CRISPR/Cas9 Technology
Source: Front Plant Sci. 2019 Nov 22;10:1446. doi: 10.3389/fpls.2019.01446 (PMC6882952; doi:10.3389/fpls.2019.01446)
Supplement: Supplementary file 1 [file DataSheet_1.docx]

**Supplemental Materials**

**Supplementary Figure 1. T-DNA region of the** **pBSE401 plasmid.** LB, T-DNA left border; polyA, 35S polyA terminator; *bar*, selective marker gene; 35S, CaMV 35S promotor; Cas9, codon-optimized Cas9; sgRNA, single guide RNA; U6, Arabidopsis U6 promotor; RB, T-DNA right border.

**Supplementary Figure 2. Heterozygous sequence leak of T_0_ generation.**

**Supplementary Figure 3. Nucleotide sequences alignment of *E1* mutations with wild type.** The premature translation termination codons for two types of mutations were marked by red boxes and [asterisk](javascript:;)s.

**Supplementary Figure 4. Amino acid sequence alignment of *E1* mutations with wild type.** Nuclear [location](javascript:;) signal was marked with a red box. The B3-like domain was marked with a dashed red line.

**Supplementary Figure 5. Potential off-target sequences of T_1_ transgenic plants.** WT, wild type plants. T_1_, mutant plants.

**Supplementary Figure 6. The other agronomic traits of** **mutants and wild type plants under natural condition.** (a) Comparison of plant height between mutants and wild type plants. (b) Comparison of branch number between mutants and wild type plants. (c) Comparison of node of stem between mutants and wild type plants.

**Supplementary Figure 7. Expression patterns of *E1*-*L* in WT plants and T_2_ homozygous mutants under LD and SD conditions.** The relative expression levels are showed as the mean values ± standard deviation, which was calculated from three biological replicates.
