# Supplementary figures and images for: Creation of Early Flowering Germplasm of Soybean by CRISPR/Cas9 Technology
Source: Front Plant Sci. 2019 Nov 22;10:1446. doi: 10.3389/fpls.2019.01446 (PMC6882952; doi:10.3389/fpls.2019.01446)

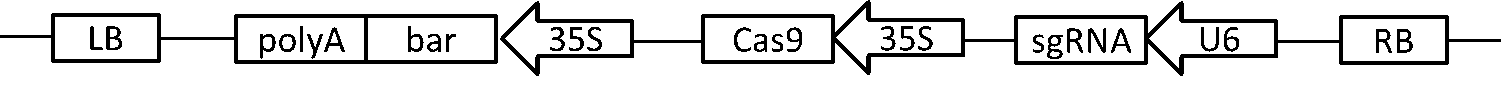

Supplement: Supplementary file 2 [file Image_1.png]

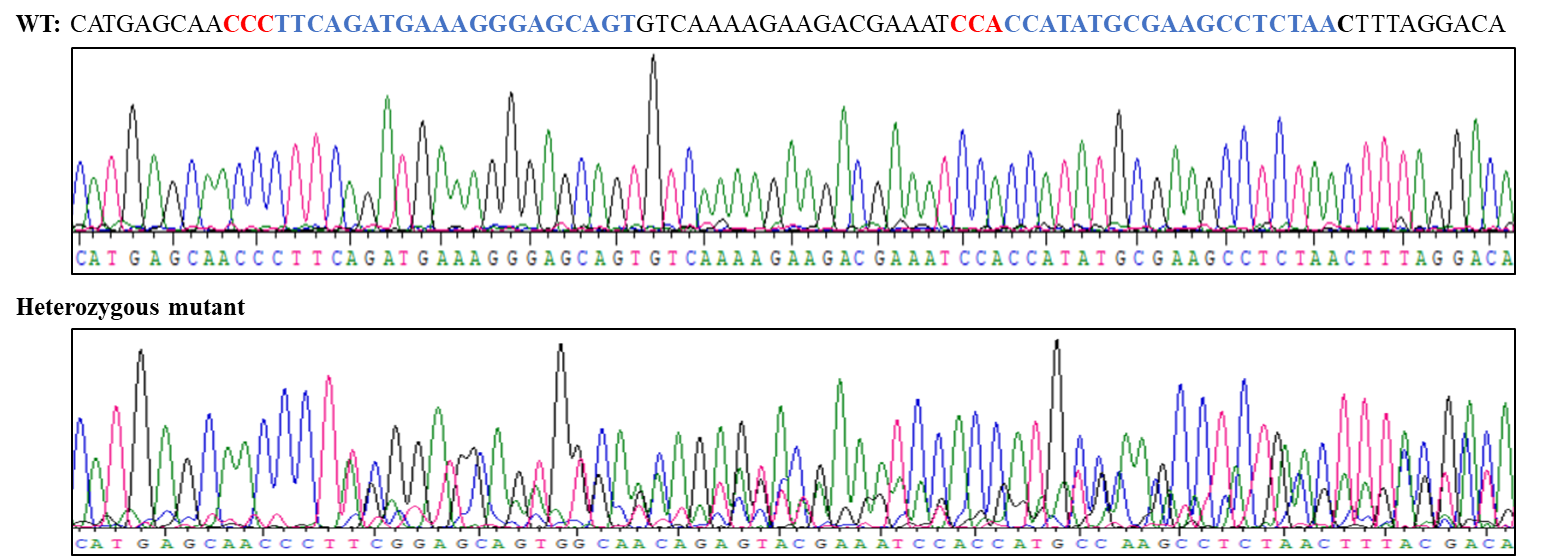

Supplement: Supplementary file 3 [file Image_2.png]

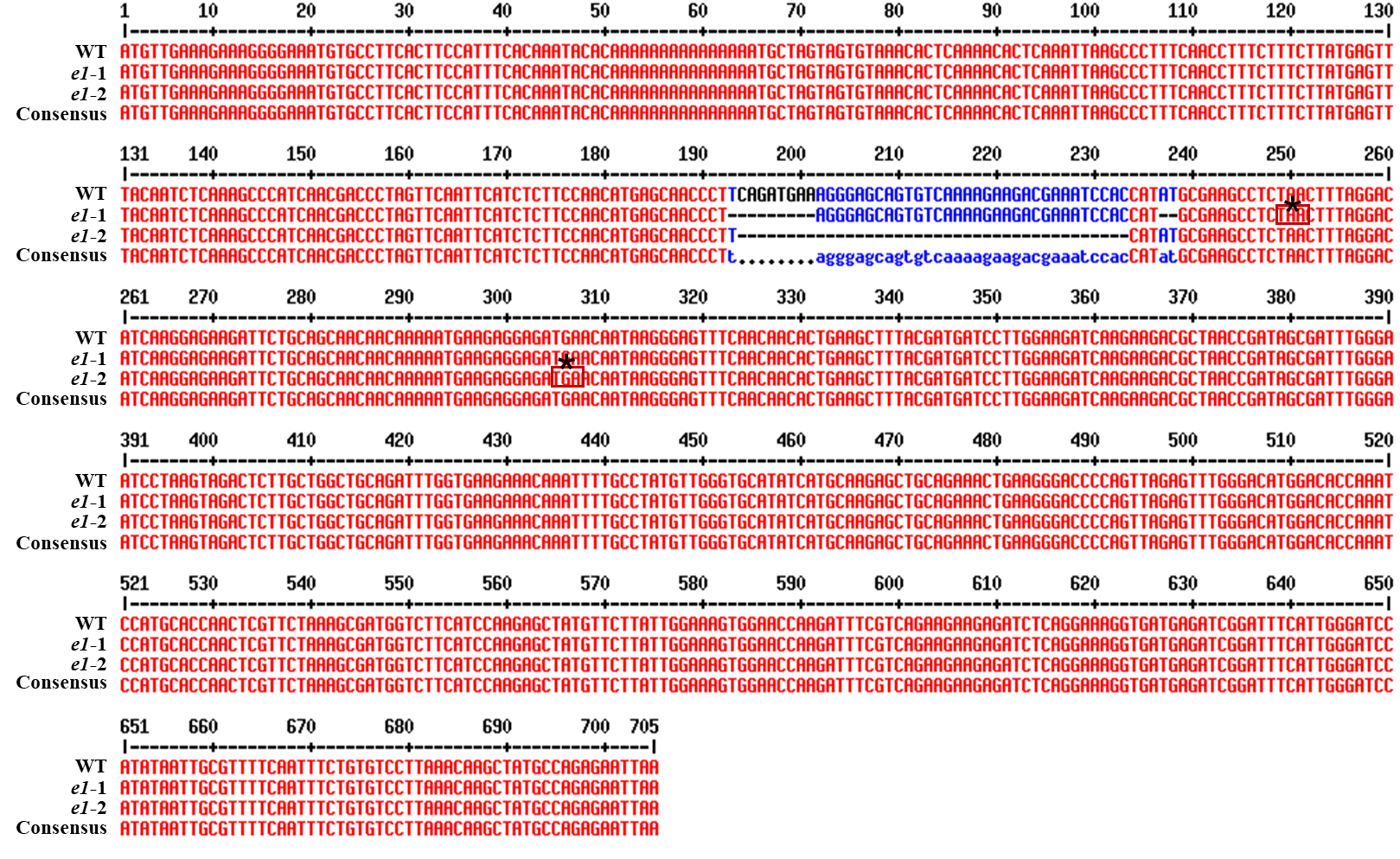

Supplement: Supplementary file 4 [file Image_3.png]

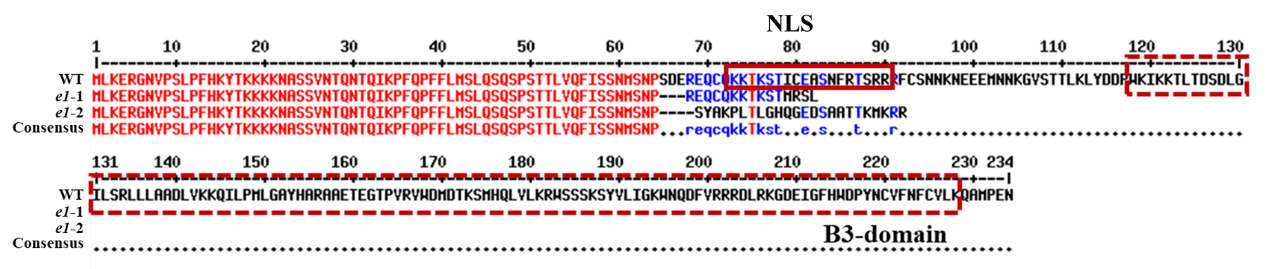

Supplement: Supplementary file 5 [file Image_4.png]

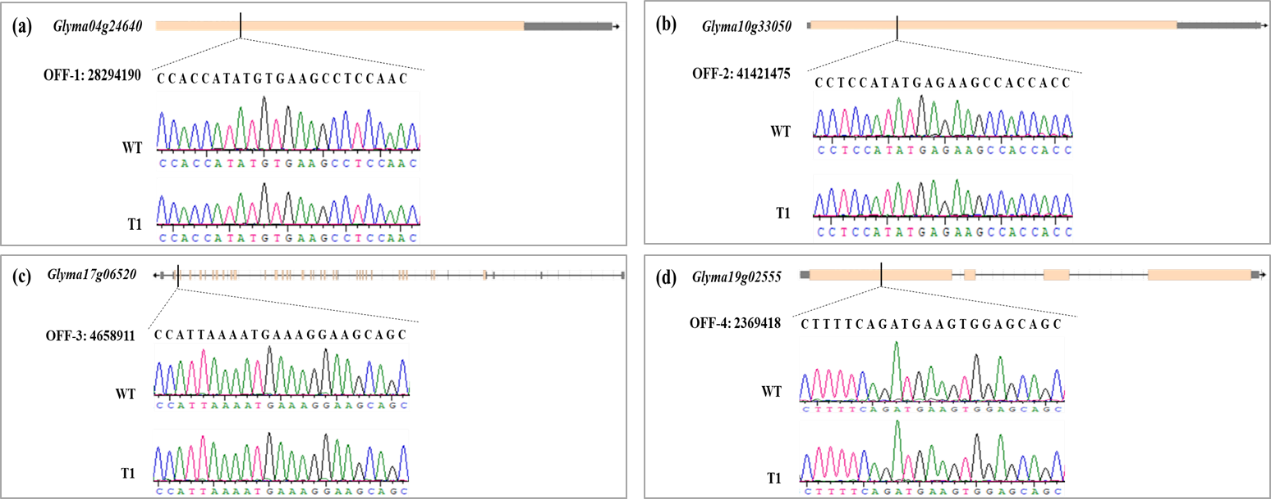

Supplement: Supplementary file 6 [file Image_5.png]

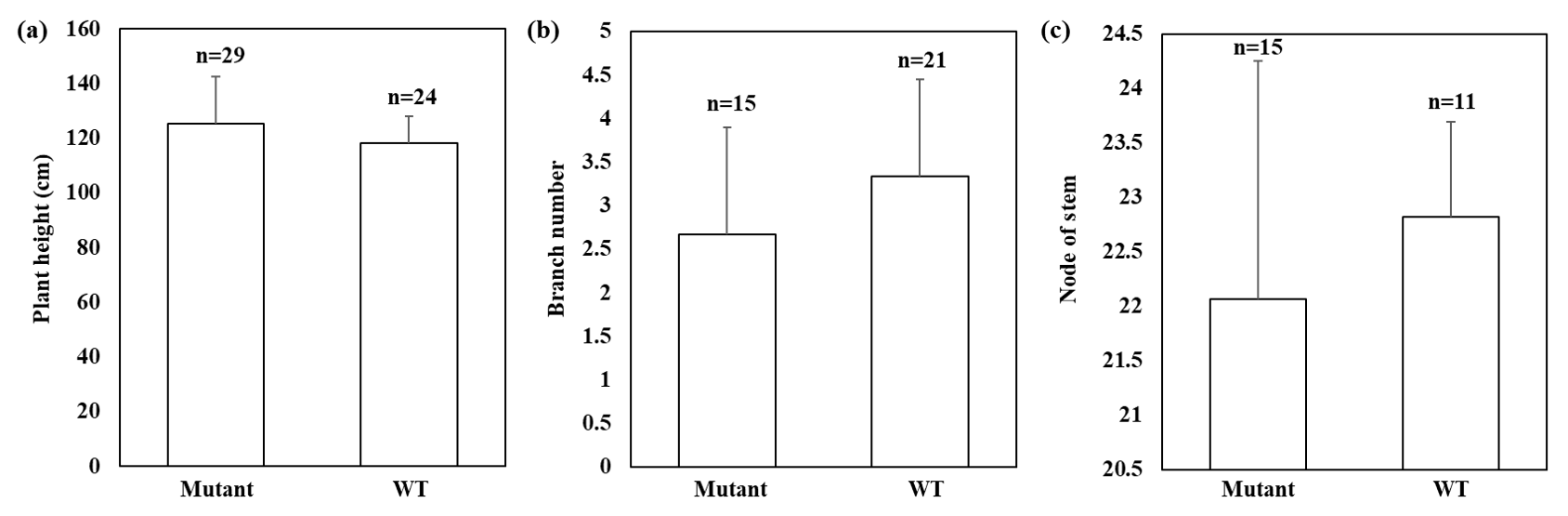

Supplement: Supplementary file 7 [file Image_6.png]

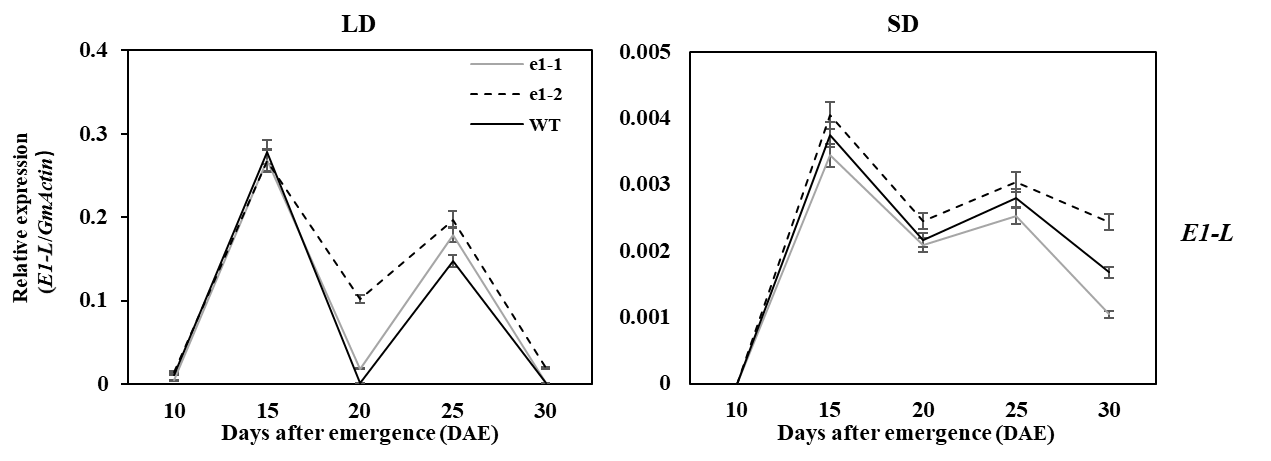

Supplement: Supplementary file 8 [file Image_7.png]
