# Supplementary material for: Creation of Early Flowering Germplasm of Soybean by CRISPR/Cas9 Technology
Source: Front Plant Sci. 2019 Nov 22;10:1446. doi: 10.3389/fpls.2019.01446 (PMC6882952; doi:10.3389/fpls.2019.01446)
Supplement: Supplementary file 9 [file Table_1.docx]

**Supplementary Table 1** Primer sequences to construct the CRISPR/Cas9 expression vector

| Primer | Primer sequence (5’→ 3’) |
| --- | --- |
| E1DT1-BSF | ATATATGGTCTCGATTGCTGCTCCCTTTCATCTGAAGTT |
| E1DT1-F0 | TGCTGCTCCCTTTCATCTGAAGTTTTAGAGCTAGAAATAGC |
| E1DT2-R0 | AACCCATATGCGAAGCCTCTAACAATCTCTTAGTCGACTCTAC |
| E1DT2-BSR | ATTATTGGTCTCGAAACCCATATGCGAAGCCTCTAACAA |

The red letters represent sgRNA sequences
