# Supplementary material for: Creation of Early Flowering Germplasm of Soybean by CRISPR/Cas9 Technology
Source: Front Plant Sci. 2019 Nov 22;10:1446. doi: 10.3389/fpls.2019.01446 (PMC6882952; doi:10.3389/fpls.2019.01446)
Supplement: Supplementary file 10 [file Table_2.docx]

**Supplementary Table 2** Primer sequences used in this study

| Primer | Primer sequence (5’→3’) | Annealing  temperature  (℃) | Purpose |
| --- | --- | --- | --- |
| E1-F | AGCCCTTTCAACCTTTCTTTCT | 59 | To amplify the target site |
| E1-R | TCCGATCTCATCACCTTTCC |  |  |
| sgRNA-F | TGTCCCAGGATTAGAATGATTAGGC | 62 | To amplify a part of sgRNA |
| sgRNA-R | AGCCCTCTTCTTTCGATCCATCAAC |  |  |
| Cas9-F | CTCCCGGATGAACACTAAGTAC | 57 | To amplify a part of Cas9 |
| Cas9-R | CAGGGTAATCTCGGTCTTGAAA |  |  |
| GmActin-F | GGTGGTTCTATCTTGGCATC | 58 | To amplify the reference gene |
| GmActin-R | GGTGGTTCTATCTTGGCATC |  |  |
| qE1-F^*^ | CACTCAAATTAAGCCCTTTCA | 58 | qRT-PCR experiment  (*E1*) |
| qE1-R^*^ | TTCATCTCCTCTTCATTTTTGTTG |  |  |
| qGmFT2a-F^*^ | ATCCCGATGCACCTAGCCCA | 60 | qRT-PCR experiment (*GmFT2a*) |
| qGmFT2a-R^*^ | ACACCAAACGATGAATCCCCA |  |  |
| qGmFT5a-F^*^ | AGCCCGAACCCTTCAGTAGGGA | 64 | qRT-PCR experiment (*GmFT5a*) |
| qGmFT5a-R^*^ | GGTGATGACAGTGTCTCTGCCCA |  |  |
| qE1-L-F^*^ | AAACACTCAAAGCCCGATCA | 58 | qRT-PCR experiment  (*E1*-*L*) |
| qE1-L-R^*^ | CCCTTGTTCATCTCCTCTTCA |  |  |
| OFF1-F | AGAAAGGGGAAATGAGCCTTC | 59 | To amplify the potential off target site 1 |
| OFF1-R | TCCGATCTCATCCCCTTTCT |  |  |
| OFF2-F | ACACTCACTCATGTTGCTCC | 60 | To amplify the potential off target site 2 |
| OFF2-R | AAGGAGGCTTGTAGATGGGT |  |  |
| OFF3-F | TAATCTTGTGGGTTGCGCAT | 60 | To amplify the potential off target site 3 |
| OFF3-R | CCACTTCTTCCCCATAGACC |  |  |
| OFF4-F | ACATATGCCCTGCCAGTTTT | 58 | To amplify the potential off target site 4 |
| OFF4-R | ACACGTGGACTGCAAACTAA |  |  |

*These primer sequences were introduced from Xia et al. (2012).
